# *ubtor* Mutation Causes Motor Hyperactivity by Activating mTOR Signaling in Zebrafish

**DOI:** 10.1007/s12264-021-00755-z

**Published:** 2021-07-26

**Authors:** Tiantian Wang, Mingshan Zhou, Quan Zhang, Cuizhen Zhang, Gang Peng

**Affiliations:** State Key Laboratory of Medical Neurobiology, Ministry of Education Frontiers Center for Brain Science, and Institutes of Brain Science, Fudan University, Shanghai, 200032 China

**Keywords:** *Ubtor*, Hyperactivity, Epilepsy, mTOR, Zebrafish

## Abstract

Mechanistic target of rapamycin (mTOR) signaling governs important physiological and pathological processes key to cellular life. Loss of mTOR negative regulators and subsequent over-activation of mTOR signaling are major causes underlying epileptic encephalopathy. Our previous studies showed that *UBTOR/KIAA1024/MINAR1* acts as a negative regulator of mTOR signaling, but whether *UBTOR* plays a role in neurological diseases remains largely unknown. We therefore examined a zebrafish model and found that *ubtor* disruption caused increased spontaneous embryonic movement and neuronal activity in spinal interneurons, as well as the expected hyperactivation of mTOR signaling in early zebrafish embryos. In addition, mutant *ubtor* larvae showed increased sensitivity to the convulsant pentylenetetrazol, and both the motor activity and the neuronal activity were up-regulated. These phenotypic abnormalities in zebrafish embryos and larvae were rescued by treatment with the mTORC1 inhibitor rapamycin. Taken together, our findings show that *ubtor* regulates motor hyperactivity and epilepsy-like behaviors by elevating neuronal activity and activating mTOR signaling.

**Supplementary Information:**

The online version contains supplementary material available at 10.1007/s12264-021-00755-z.

## Introduction

Mechanistic target of rapamycin (mTOR) is an evolutionarily conserved serine-threonine protein kinase. The mTOR signaling plays important roles in various physiological and pathological processes of cellular life [1, 2]. The loss-of-function of negative regulators of mTOR triggers behavioral abnormalities and neurological diseases by over-activating mTOR signaling [3]. For example, absence of the negative regulator tuberous sclerosis complex (TSC) 1 or TSC2 results in susceptibility to hamartoma formation and epilepsy [4, 5]. Recently, mutations in the DEP domain containing protein 5 gene (*DEPDC5*) have been identified as a crucial genetic cause of focal epilepsy [6]. DEPDC5 forms the mTORC1 inhibitory complex GATOR1 with NRPL2 and NRPL3 to block the activation of mTORC1 [7]. In a mouse model, adults with conditional knockout (cKO) of *Depdc5* exhibit cortical defects and increased susceptibility to epilepsy [8]. And *depdc5* knockdown in the zebrafish model leads to early-onset phenotypic features associated with mTOR-dependent motor and neuronal hyperactivity [9]. Nevertheless, the molecular functions of newly-identified negative regulators of mTOR in the occurrence and development of neurological diseases remain elusive.

*Ubtor* is an unannotated gene found in vertebrate species only. The protein encoded by *Ubtor* is highly conserved in vertebrates [10]. Early studies listed *Ubtor* as a gene that is down-regulated or mutated in tumor tissues [11, 12]. Our previous study showed that *Ubtor* is a negative regulator of mTOR signaling, and loss of *Ubtor* promotes neurite and cellular growth [10]. It has also been shown that *Ubtor/MINAR1* plays roles in angiogenesis and breast cancer [13]. However, the *in vivo* function of *Ubtor* remains largely unknown.

In this study, we examined the phenotypic features of *ubtor* mutant zebrafish.

## Materials and Methods

### Zebrafish Maintenance and Ethics Statement

Experiments were performed on AB, Tg(UAS:EGFP), Tg(*ubtor*:GAL4FF), and *ubtor* mutant lines. The AB line was acquired from the University of Oregon Zebrafish Facility; the Tg(UAS:EGFP) line was a gift from Dr. K Kawakami [14]; the Tg(*ubtor*:GAL4FF) line was generated by an enhancer trap screen, and the generation of the *ubtor* mutant line was as previously described [10]. All zebrafish were maintained at 28.5 °C under 14 h light/10 h dark cycles. All animal studies were carried out according to the National Research Council (NRC) Guide for the Care and Use of Laboratory Animals and the Fudan University Regulations on Animal Experiments (Approval #170223-101).

### Drug Treatments

PTZ and rapamycin were from Sigma (St. Louis, USA). PTZ stock was freshly prepared in nuclease-free water. Rapamycin stock was dissolved in dimethyl sulfoxide (DMSO) and stored at −20 °C. All drugs were diluted in embryonic medium to working concentrations indicated in the text. Zebrafish embryos were exposed to the drugs by soaking and kept in the same solution during behavioral testing.

### Motor Activity Analysis

To assess coiling behavior inside the chorion, 28-h post-fertilization (hpf) zebrafish embryos in their chorion were acclimated to room temperature for 30 min, then transferred into a 1.2-mm wide slot in 2.5% agarose, submerged in embryonic medium, and imaged using a camera (Point Grey, BC, Canada) at 30 Hz. Recorded videos were inspected and the coiling movements of embryos were manually counted.

To assess coiling behavior outside the chorion, embryos at 10 hpf were gently dechorionated using tweezers. A custom-made 5 × 4 grid was laser-cut into a 70 mm × 50 mm × 2 mm (length × width × thickness) acrylic plate to hold the embryos. A single 28-hpf zebrafish was placed in each grid well, and the coiling behaviors were recorded at 30 Hz.

To assess PTZ-induced motor activity, zebrafish larvae at 4 dpf were placed in a 24-well plate, with a single zebrafish and 1 mL embryonic medium in each well. Before PTZ induction, all larvae were adapted to the new environment for 10 min. PTZ stock was added to each well and the larval movements were recorded immediately for 10 min. The recorded videos were processed and analyzed with custom-written MATLAB scripts.

### *In situ* Hybridization (ISH)

Whole-mount *in situ* hybridization of zebrafish embryos or dissected brain tissue was performed as described previously [15]. Digoxigenin-labeled antisense RNA probes were detected with the alkaline phosphatase-conjugated digoxigenin antibody Fab fragment (1:5000, Roche, Basel, Switzerland) and the alkaline phosphatase substrate NBT/BCIP (1:50, Roche). Probe constructs were generated by RT-PCR using the primers listed in Table 1, followed by standard DNA cloning methods.

**Table 1:** Primers used to generate probe constructs used in this study.

Probe	Sequence
*ubtor*-forward	5′-CTTCCCAAAATCAACAATCAAAACAAC
*ubtor*-reverse	5′-GAACACCTGCTCCACTAGGTAG
*c-fos*-forward	5′-CCCGAGCTCTTACCCCAAAA
*c-fos*-reverse	5′-GCAATGGTTAGGCAGCACAC
*vglut*2*a*-forward	5′-AGGGAGCCTGCTGGTTTTAG
*vglut*2*a*-reverse	5′-CGACAGCCAACACTAGAAATGA
*evx2*-forward	5′-TTTGGGAACCACCACAACGA
*evx2*-reverse	5′-CTGGGCGTACACTGGAATGA
*slc6a5*-forward	5′-CAAGAAAATGTCATGCGAAGGGTA
*slc6a5*-reverse	5′-CCACATACGGGAATGTTGCTG
*pax2b*-forward	5′-TAACACGCAAATGGACGGGA
*pax2b*-reverse	5′-CGCTGGACGATAAACTGGGT
*gad2*-forward	5′-TCCAGTCTTCGTGCTGTTGG
*gad2*-reverse	5′-AGGGACACGGGAGTAACAGG

### Calcium Imaging

Zebrafish embryos at 28 hpf were mounted in 1.8% low-melting agarose and the agarose around the tail was carefully removed. Images were acquired at 20 Hz using a spinning disk microscope (Andor, Belfast, Northern Ireland). The recorded images were processed with the NoRMCorre and CaImAn packages to extract neuronal Ca^2+^ signals [16, 17]. Then, the MLspike method [18] was applied to extract spiking activity from the Ca^2+^ signals.

### Total RNA Extraction and Reverse Transcription

The zebrafish embryos were placed in an ice-water bath, anaesthetized, and transferred into a 1.5-mL Eppendorf tube. 300 μL of lysis buffer containing β-mercaptoethanol was added and the embryos were ground using a plastic pestle (Sangon Biotech, Shanghai, China). Total RNA extraction from the spinal tissue included the muscle tissue surrounding the spinal cord. The RNA of the samples was extracted using a total-RNA extraction kit (Sangon Biotech). The reverse transcription reactions were conducted using a PrimeScript RT reagent kit (TaKaRa, Shiga, Japan).

### Quantitative Real-Time PCR (qRT-PCR)

qRT-PCR was performed as previously described [15]. The results were normalized to β-actin and calculated by the normalized *C*_T_ values (2^−ΔΔCt^). The primers used in qRT-PCR assays are listed in Table 2.

**Table 2:** Primers used in qRT-PCR assays.

Primer	Sequence
*β-actin*-forward	5′-GCTAGTGCGGAATATCATCTGC
*β-actin*-reverse	5′-TCCCATACCAACCATGACACC
*c-fos*-forward	5′-AGCAGACGAGCAAGGAAATACAAGAC
*c-fos*-reverse	5′-ATGAAGAGATCGCCGTGACAGTTG
*vglut*2*a*-forward	5′-CAAGACTGGCGGCAAACAGGG
*vglut*2*a*-reverse	5′-GACAGCCCCAGCATAGGAACC
*evx2*-forward	5′-CACCACAACGACAACACTTCC
*evx2*-reverse	5′-GAATTGCTGTCCGAGAAACCTTTA
*slc6a5*-forward	5′-ACCTATGTCAGCCCTAGCGA
*slc6a5*-reverse	5′-GACCAACACCACATACGGGA
*pax2b*-forward	5′-CAGGCGAGTGAATACAGCCT
*pax2b*-reverse	5′-TTCGCTCCCAGGAACCATTC
*gad2*-forward	5′-GCGCTACCTGGAGGACAAAG
*gad2*-reverse	5′-CAGGTTTTGGTGCGGTAAGT

### Western Blot

Zebrafish tissues were lysed in 1× SDS sample buffer (50 mmol/L Tris-HCl pH 6.8, 2% SDS, 0.01% bromophenol blue, 10% glycerol, and 100 mmol/L dithiothreitol) and boiled at 98 °C for 10 min. Proteins were resolved by 10% SDS-PAGE and transferred onto nitrocellulose membranes, which were incubated overnight with primary antibodies (anti-p-S6, Cell Signaling Technology, Boston, USA, cat#4858S; anti-RPS6, Proteintech, Chicago, USA, cat#14823-1-AP; and anti-β-actin, Proteintech, cat#66009-1-Ig), washed three times, and incubated with Horseradish Peroxidase (HRP)-conjugated secondary antibodies (anti-mouse IgG, Cell Signaling Technology, cat#7076S; and anti-rabbit IgG, Cell Signaling Technology,cat#7074S). Immuno-signals were generated by chemiluminescence (Cell Signaling Technology, cat#12757), scanned by a FluorChem E system (ProteinSimple, Silicon Valley, USA), and analyzed using ImageJ software.

### Immunofluorescence Analysis

The immunofluorescence analysis was carried out as previously described ^[10]^. In brief, zebrafish were fixed with 4% paraformaldehyde (PFA) for 2 h at room temperature. The fixed embryos were washed with PBS and then post-fixed with acetone at −20 °C for 20 min, blocked, and incubated overnight with primary antibodies at −4 °C. The embryos were then washed and incubated with secondary antibodies.

### Statistical Analysis

Statistical analysis was performed with GraphPad Prism 7. Data are shown as the mean ± SEM. Student’s *t*-tests were conducted when appropriate. *P* <0.05 was considered statistically significant: *P <0.05, **P <0.01, and ***P <0.001.

## Results

### *ubtor* is Expressed in Spinal Interneurons in Zebrafish Embryos

**Fig. 1:**
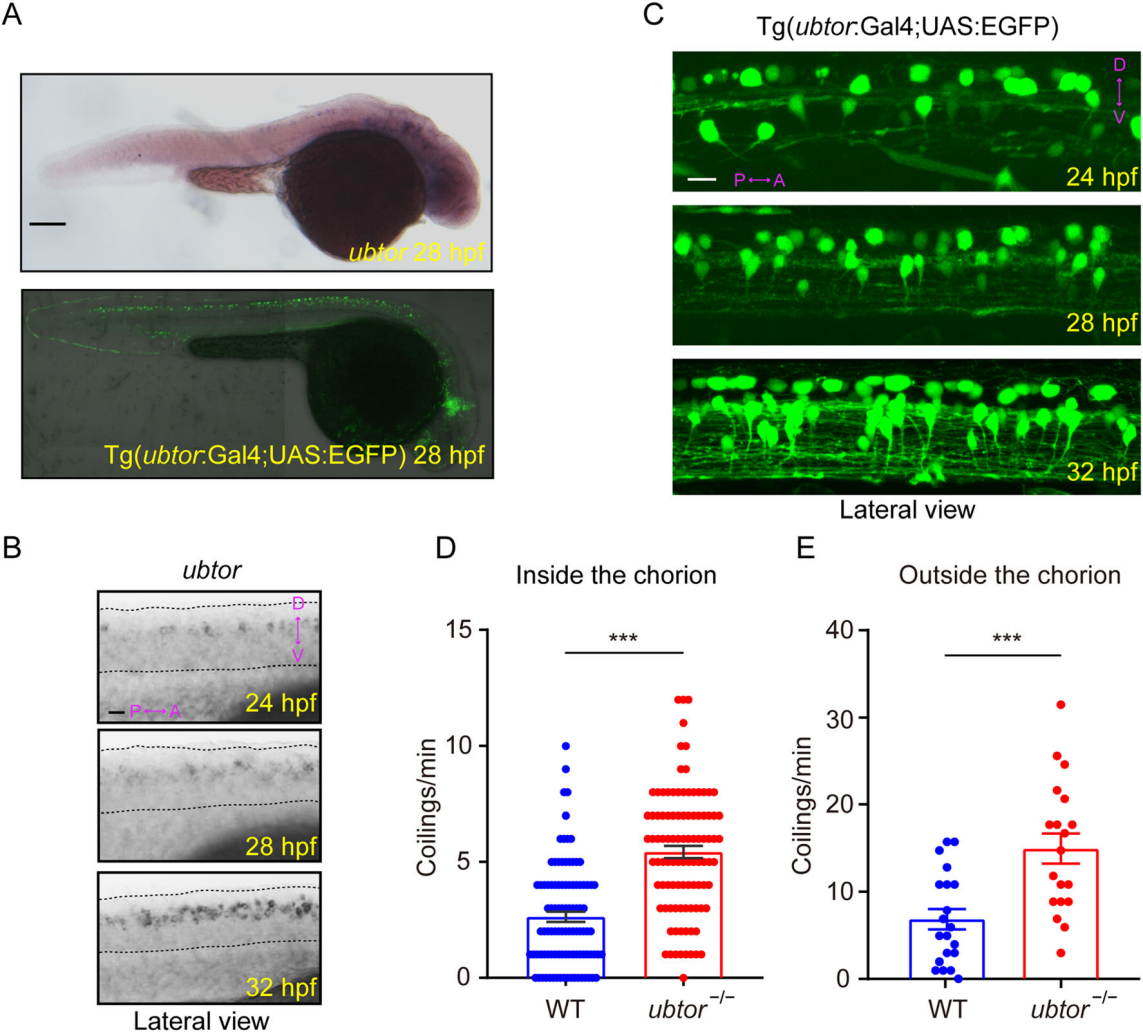
*ubtor* is expressed in spinal interneurons and *ubtor* mutants show increased spontaneous coiling. **A** *ubtor* expression (upper) and EGFP expression (lower) in 28-hpf Tg(*ubtor*:GAL4;UAS:EGFP) zebrafish embryos (scale bar, 250 μm). **B** Expression and distribution of the *ubtor* gene in zebrafish embryo spinal cord tissues at 24, 28, and 32 hpf (dotted lines, spinal cord tissue from hindbrain to somite 20. Scale bar, 60 μm. **C** EGFP expression in the spinal cord tissue of embryos at somites 4–10 of the Tg(*ubtor*:GAL4;UAS:EGFP) line (scale bar, 20 μm). **D** Frequency of coiling movements of embryos inside the chorion of 28-hpf *ubtor* mutants and the wild-type (results from five biological repeats, N_WT_ = N_*ubtor*_^−/−^ = 126, *t*_198_ = 8.097). **E** Frequency of coiling movements outside the chorion of 28-hpf *ubtor* mutants and the wild-type (two biological repeats, N_WT_ = N_*ubtor*_^−/−^ = 20, *t*_37_ = 3.914). Values are represented as the mean ± SEM in **D** and **E**. ***P <0.001; P, posterior; A, anterior; D, dorsal; V, ventral.

We performed *in situ* hybridization (ISH) analysis of 28-hpf zebrafish embryos and found that *ubtor* mRNA was specifically enriched in the spinal cord and hindbrain, with little to no signal detected in other structures (Fig. 1A). To determine the timeline of *ubtor* expression in the spinal cord during development, we analyzed the presence of *ubtor* transcripts by ISH at several stages of embryonic development: 24, 28, and 32 hpf. Previous studies have shown that zebrafish and mammalian spinal cord neurons have a similar distribution, and different types of neurons are stereotypically arranged along the dorsoventral axis [19] (Fig. S1A). We found that the *ubtor* gene was already specifically expressed in interneurons along the dorsoventral axis in the spinal cord of embryos at 24 hpf, but not on the ventral side of the cord (Fig. 1B). EGFP expression patterns in the transgenic line Tg(*ubtor*:Gal4;UAS:EGFP) were similar to the results of *ubtor* ISH in the spinal cord (Fig. 1C and S1). Meanwhile, the expression pattern of *ubtor* in spinal interneurons consistently increased with time over the course of development. To further examine the distribution of *ubtor*-expressing interneurons in the spinal cord, we carried out double-labeling by *ubtor:*GFP and neural markers (*slc6a5/glyT2* and *gad2*) by using immunofluorescence and ISH assays. Extensive overlap of *ubtor*:GFP and *slc6a5/glyT2* was observed, and some overlap of *ubtor*:GFP and *gad2* was seen in the spinal cord of the wild-type at 28 hpf (Fig. S1B, C). These results indicate that the neurons expressing *ubtor* are mainly located in the middle of the spinal cord and *ubtor* may play a role in spinal interneuron functions.

### *ubtor* Mutants Show Hyperactive Spontaneous Movement

Spinal interneurons have been identified in the regulation of movement behaviors and are key to connection and integration in the spinal circuits of zebrafish [20]. Zebrafish embryos exhibit early stereotyped motor activity starting at 17 hpf, showing repetitive twitches (tail flicks) and coils (complete rotations) at regular intervals, the frequencies varying with age [21]. Spontaneous movement frequency is controlled by a synchronized, bilateral spinal circuit [22]. Because *ubtor* is expressed in spinal interneurons at early developmental stages, *ubtor* may participate in the spinal interneuron-related movement. To test this hypothesis, we examined the spontaneous coiling behavior in *ubtor* mutant embryos. Our results showed that there was no significant difference of coiling behavior in 21-hpf wild-type and *ubtor* mutant embryos (*ubtor* mutants: 10.38 ± 1.307 coils/min *vs* control: 9.875 ± 1.121 coils/min, *P* = 0.774, Fig. S2). Importantly, *ubtor* mutant embryos exhibited a hyperactive phenotype inside the chorion at 28 hpf (*ubtor* mutants: 5.43 ± 0.265 coils/min *vs* control: 2.63 ± 0.222 coils/min, *P* <0.001, Fig. 1D). A similar phenomenon was present in *ubtor* mutant embryos outside the chorion at 28 hpf (*ubtor* mutants: 15.21 ± 1.763 coils/min *vs* control: 6.95 ± 1.195 coils/min, *P* <0.001, Fig. 1E). Taken together, these data show that *ubtor* is expressed in spinal interneurons and that its loss-of-function causes hyperactive spontaneous movement in zebrafish embryos.

### *ubtor* Mutation Increases Activity in Spinal Interneurons Without Affecting their Development

**Fig. 2:**
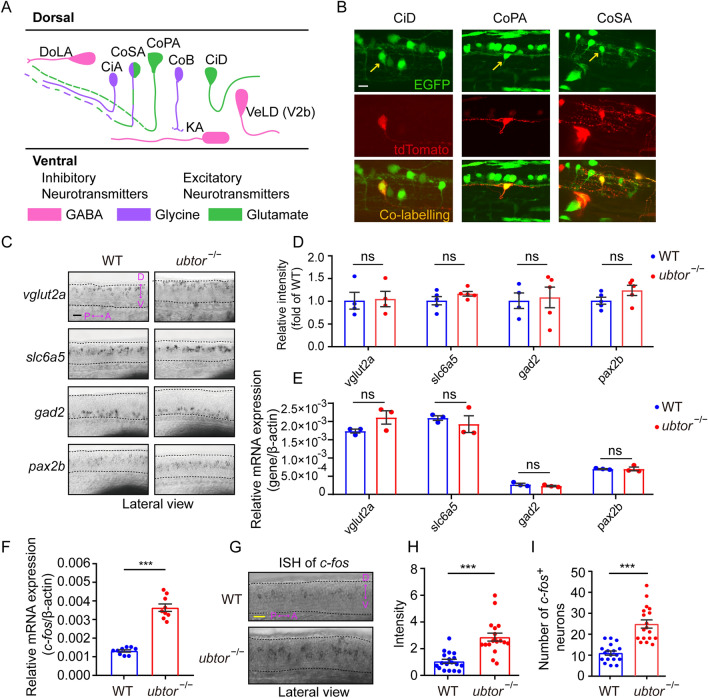
*ubtor* mutants show increased activity in spinal interneurons. **A** Schematic of different types of spinal interneurons (modified from [20]). **B** Mosaic labeling of spinal interneurons by tdTomato in 28-hpf Tg(*ubtor*:GAL4;UAS:EGFP) transgenic embryos (scale bar, 20 μm). Arrows indicate mosaic labelling interneurons. **C** Expression and distribution of spinal interneuron markers in spinal somites 4–10 (scale bar, 60 μm). **D** Quantification of relative intensity of spinal interneuron markers (*vglut*2*a*: N_WT_ = N_*ubtor*_^−/−^ = 5, *t*_6_ = 0.150; *slc6a5*: N_WT_ = N_*ubtor*_^−/−^ = 5, *t*_8_ = 1.462; *gad2*: N_WT_ = N_*ubtor*_^−/−^ = 5, *t*_7_ = 0.2453; *pax2b*: N_WT_ = N_*ubtor*_^−/−^ = 5, *t*_8_ = 1.662). **E** RT-qPCR analyses of expression levels of spinal interneuron markers in the trunk tissues of 28-hpf embryos (three biological repeats, each marker: N_WT_ = N_*ubtor*_^−/−^ = 75). **F** RT-qPCR analysis of *c-fos* expression levels in the trunk tissues of 28-hpf embryos (three biological repeats, N_WT_ = N_*ubtor*_^−/−^ = 75, *t*_16_ = 10.99). **G** Expression and distribution of *c-fos* in spinal somites 4–10 (scale bar, 40 μm). **H** Intensity of *c-fos*-labeled neurons in spinal cord (two biological repeats, N_WT_ = N_*ubtor*_^−/−^ = 18, *t*_34_ = 5.348). **I** Numbers of *c-fos* labeled neurons in spinal cord (two biological repeats, N_WT_ = N_*ubtor*_^−/−^ = 18, *t*_34_ = 6.324). β-actin served as the internal control. Values are represented as the mean ± SEM. In **D**–**F**, **H**, and **I**, ***P <0.001. Abbreviations: DoLA, dorsal longitudinal ascending; CiA, circumferential ascending; CoSA, commissural secondary; CoPA, commissural primary ascending; CoB, commissural bifurcating; KA, Kolmer-Agduhr; CiD, circumferential descending; VeLD, ventral longitudinal descending.

The cell bodies of spinal interneurons (comprising GABAergic, glycinergic, and glutamatergic neurons, Fig. 2A, modified from [20]) are located in the central nervous system and receive signals from sensory (afferent) neurons, which are then transmitted to the motor (efferent) neurons and generate corresponding motor behaviors. To determine the types of interneurons in the spinal cord that express the *ubtor* gene, we used a mosaic single-cell labeling method. A mixture of a Tol2-UAS:tdTomato plasmid and Tol2-transposase mRNA was microinjected into one-cell stage Tg(*ubtor*:Gal4; UAS:EGFP) embryos to sparsely label spinal neurons with the red tdTomato signal. The results showed that tdTomato successfully labeled CiD (circumferential descending), CoPA (commissural primary ascending), and CoSA (commissural secondary ascending) glutamatergic neurons and CoLA (commissural longitudinal), CoB (commissural bifurcating), and CoSA glycinergic neurons. These labeled glutamatergic and glycinergic neurons were co-labeled with EGFP in transgenic fish, indicating that the neurons expressing *ubtor* are glutamatergic or glycinergic (Figs S2B and S3).

To determine if *ubtor* mutation alters the development of spinal neurons, we examined the expression profiles of known interneuron markers using *in situ* hybridization. Compared with wild-type controls, there were no significant differences in the expression and distribution patterns of the *vglut*2*a* (glutamatergic marker), *slc6a5*, *pax2b* (glycinergic markers), and *gad2* (GABAergic marker) genes in the spinal cord of the *ubtor* mutant (Fig. 2C–E). These results suggest that the *ubtor* gene mutation does not significantly affect the development of spinal interneurons in zebrafish.

Studies have shown that abnormal neuronal activity is one of the main causes of epilepsy. *C-fos* is an immediate-early gene that is widely used as a molecular marker of neuronal activity [23, 24]. It is transiently induced in the central nervous system during seizures [25]. To determine whether the hyperactive phenotypic features of *ubtor* mutant larvae were correlated with increased neuronal activity, we assessed the expression of the *c-fos* gene in the spinal cord neurons of 28-hpf embryos. Using the qRT-PCR and ISH methods, we found significantly increased *c-fos* expression levels in spinal interneurons in *ubtor* mutant larvae compared with their wild-type siblings (Fig. 2F, G). Meanwhile, the number of spinal interneurons expressing *c-fos* was also significantly increased (Fig. 2H, I).

**Fig. 3:**
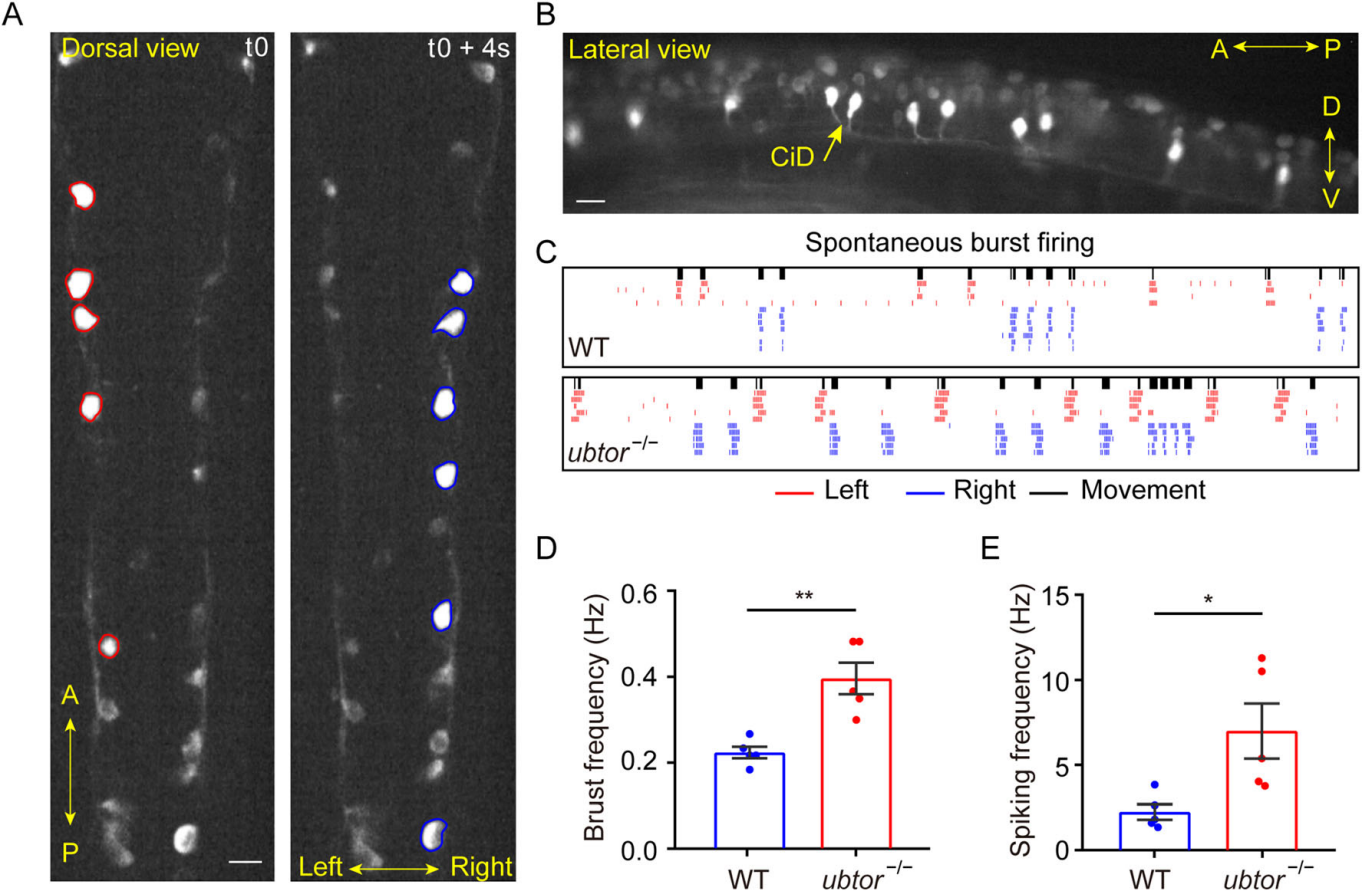
Calcium imaging of spinal interneurons. **A** Representative Ca^2+^ signals of spinal cord interneurons. At t0 (an arbitrary time point in the video sequence), interneurons on the left side are firing, followed by interneurons on the right side 4 s later (dorsal view; scale bar, 40 μm). **B** Firing interneurons are CiD interneurons; the Ca^2+^ signal lights up the soma and the descending axons of CiD interneurons during a burst (lateral view, scale bar, 40 μm). **C** Rasters of correlated muscle movements and neuronal bursts (black bars, muscle movements; red and blue lines, left and right interneuron spikes estimated by the MLspike method). **D**, **E** Quantitative analysis of neuronal burst frequency (N_WT_ = N_ubtor_^−/−^ = 5, *t*_8_ = 4.396; **D** and spike frequency (N_WT_ = N_*ubtor*_^−/−^ = 5, *t*_8_ = 2.821; **E** of spinal interneurons. Values are represented as the mean ± SEM in **D** and **E**. *P <0.05, **P <0.01.

We further investigated the spinal neuron activity by Ca^2+^ imaging. Because early zebrafish embryos are transparent, Ca^2+^ imaging has been used in these models to study neural activity, including several models of epilepsy [26, 27]. We bred a Tg(*ubtor*:Gal4;UAS:GCaMP5HS) transgenic tool line into the *ubtor* mutant and found prominent changes in the Ca^2+^ signals in CiD-type spinal interneurons both in the *ubtor* mutant and the wild-type (Fig. 3A, B). Importantly, the frequency of burst and spike activity in the CiD interneurons was higher in *ubtor* mutant embryos, as inferred from MLSpike analysis [18] (Fig. 3C–E). This suggested that the activity of CiD neurons was higher in the *ubtor*-mutated group than in the wild-type control group, consistent with up-regulation of the *c-fos* gene in spinal neurons with *ubtor* depletion. Collectively, these data demonstrate that the loss-of-function of *ubtor* enhances the activity of spinal interneurons and up-regulates early spontaneous movement in zebrafish.

### Spinal Interneurons Exhibit mTORC1 Hyperactivation in *ubtor* Mutant Embryos

**Fig. 4:**
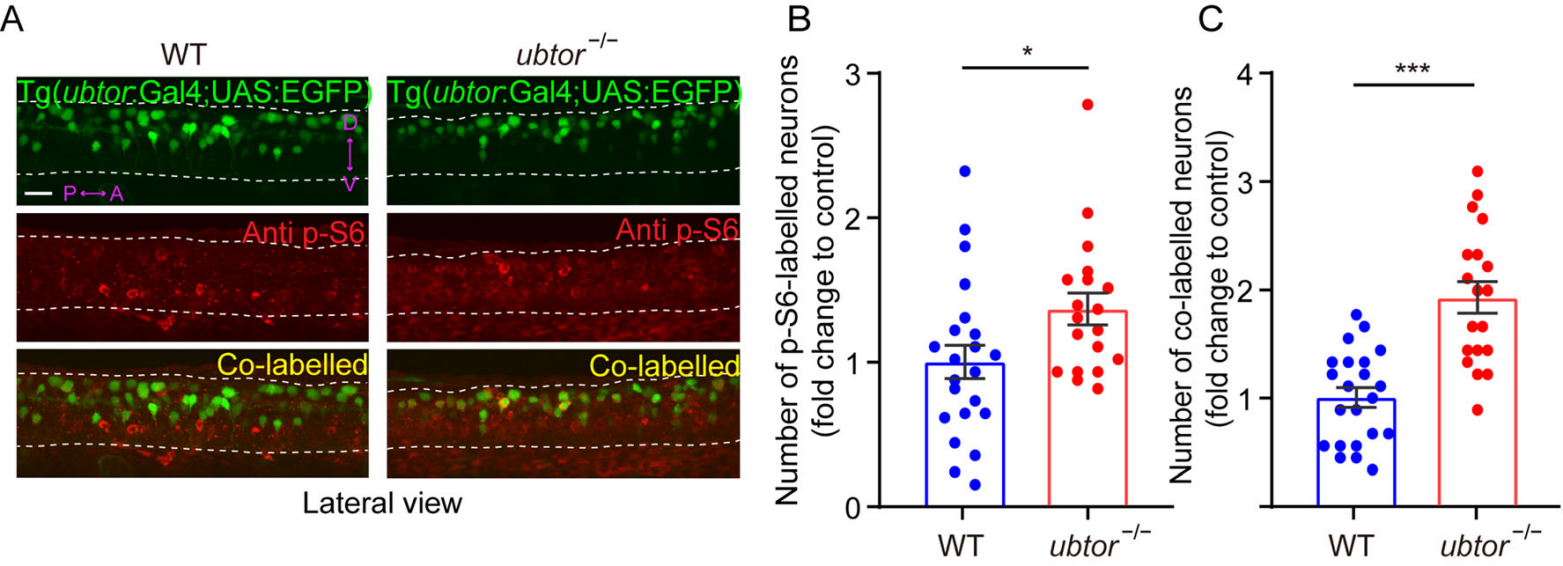
mTORC1 hyperactivation in the spinal interneurons of *ubtor* mutant embryos. **A** p-S6 staining and EGFP signals in 28-hpf embryos (dotted lines outline spinal somites 4–10; scale bar, 40 μm). **B** Numbers of p-S6-labelled neurons in the spinal cord of 28-hpf embryos (three biological repeats, N_WT_ = 22, N_*ubtor*_^−/−^ = 19, *t*_39_ = 5.538). **C** Numbers of co-labeled p-S6/EGFP neurons in the spinal cord of 28-hpf embryos (three biological repeats, N_WT_ = 22, N_*ubtor*_^−/−^ = 19, *t*_39_ = 2.265). Values are represented as the mean ± SEM in **B** and **C**. *P <0.05, ***P <0.001.

It has been reported that up-regulation of the mTOR signaling pathway affects motor behaviors and neuronal excitability [28]. Our previous studies showed that, in both mammalian cells and zebrafish tissues, depletion of *Ubtor* up-regulates activity in the mTORC1 pathway [10]. Therefore, we speculated that the increase in spontaneous movement and up-regulated activity of spinal interneurons in 28-hpf zebrafish may be regulated by the mTORC1 pathway. We assessed the phosphorylation state of RPS6, a downstream substrate of mTORC1, by immunofluorescence in *ubtor* mutants in the Tg(*ubtor*:Gal4;UAS:EGFP) background. In this background, EGFP fluorescence represents the expression of the endogenous *ubtor* gene, p-S6 (phosphorylated RPS6 protein) represents the activation status of the mTORC1 pathway, and EGFP neurons co-labeled with p-S6 represent the activation of the mTORC1 pathway in *ubtor* neurons. These results showed that, compared with the wild-type control, the number of p-S6-positive spinal interneurons in the *ubtor* mutant increased significantly at 28 hpf (Fig. 4A, B), as did the number of neurons co-labeled with EGFP and p-S6 (Fig. 4C). We further found that the distribution of p-S6-positive neurons was similar in the controls and the *ubtor* mutants (Fig. S4A). Second, when we compared the number of p-S6-positive and EGFP-negative neurons between the control and the *ubtor* mutants, we found no significant difference (Fig. S4B). These results suggest that disruption of the *ubtor* gene likely up-regulates mTORC1 pathway activity in neurons expressing *ubtor* in the spinal cord.

### Rapamycin Normalizes the Increased Movement and Neuronal Activity in *ubtor* Mutants

**Fig. 5:**
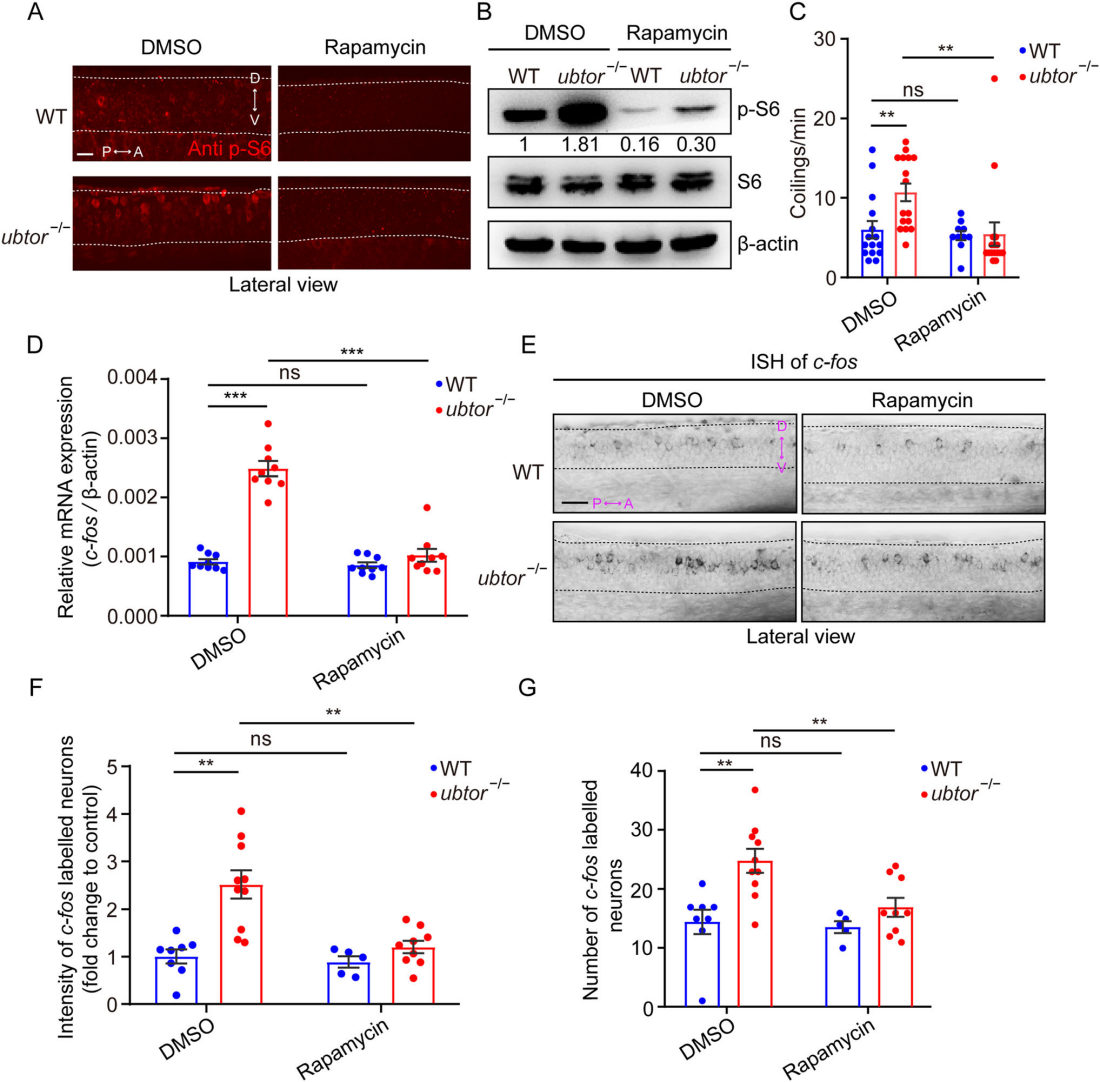
Rapamycin normalizes spontaneous movements and the activity of spinal interneurons in 28-hpf *ubtor* mutant embryos. **A** p-S6 staining in the spinal cord of 28-hpf embryos treated with DMSO (solvent) or 100 nmol/L rapamycin (dashed lines, spinal somites 4–10; scale bar, 40 μm). **B** Immunoblotting for p-S6 and S6 protein in 28-hpf embryos treated with DMSO or 100 nmol/L rapamycin. Quantified p-S6 protein levels are indicated. **C** Frequency of coiling movements outside the chorion of 28-hpf embryos treated with DMSO or rapamycin (two biological repeats, DMSO: N_WT_ = N_*ubtor*_^−/−^ = 16, *t*_29_ = 2.974; rapamycin: N_WT_ = N_*ubtor*_^−/−^ = 16, *t*_29_ = 0.189). **D** RT-qPCR analysis of *c-fos* mRNA levels in 28-hpf embryos treated with DMSO or rapamycin (three biological repeats, DMSO: N_WT_ = N_*ubtor*_^−/−^ = 75, *t*_16_ = 11.57; rapamycin: N_WT_ = N_*ubtor*_^−/−^ = 75, *t*_16_ = 1.389). **E** Expression and distribution of *c-fos* using *in situ* hybridization assays in 28-hpf embryos treated with DMSO or 100 nmol/L rapamycin (spinal somites 4–10; scale bar, 40 μm). **F**, **G** Relative intensity (DMSO: N_WT_ = 8, N_*ubtor*_^−/−^ = 10, *t*_16_ = 4.246; rapamycin: N_WT_ = 6, N_*ubtor*_^−/−^ = 9, *t*_12_ = 1.586; **F**) and number (DMSO: N_WT_ = 8, N_*ubtor*_^−/−^ = 10, *t*_16_ = 3.520; rapamycin: N_WT_ = 6, N_*ubtor*_^−/−^ = 9, *t*_12_ = 1.453; **G**) of *c-fos* labeled spinal interneurons in 28-hpf embryos treated with DMSO or rapamycin. β-actin served as thein ternal control. Values are represented as the mean ± SEM in **C**, **D**, **F**, and **G**. **P <0.01, ***P <0.001.

Rapamycin is a macrolide antibiotic and a specific inhibitor of the mTORC1 complex; it has been used in the field of anti-epileptic research [29]. Our study further investigated the effect of rapamycin on mTORC1 pathway activity in early embryonic tissues. The zebrafish embryos were dechorionated and treated with rapamycin at 10 hpf. After the embryos reached 28 hpf, immunofluorescence and Western blot experiments were performed to assess the activity of the mTORC1 pathway. Our results showed that rapamycin decreased the number of p-S6-positive spinal interneurons (Fig. 5A) and p-S6 protein levels (Fig. 5B) both in *ubtor* mutants and wild-type controls. Importantly, rapamycin treatment rescued the hyperactive movements in *ubtor* mutant embryos (Fig. 5C).

Our results above showed that the spontaneous motor enhancement of *ubtor* mutants at 28 hpf is regulated by up-regulation of the mTORC1 pathway, along with the enhanced activity of spinal interneurons. Studies have shown that the mTORC1 pathway is involved in regulating the excitability of neurons [30]. In order to assess whether the mTORC1 pathway increases early spontaneous movement by regulating the neuronal activity of spinal interneurons in zebrafish, we examined the effect of rapamycin treatment on the activity of spinal neurons. The results showed that rapamycin treatment significantly decreased the *c-fos* mRNA levels in the spinal interneurons of the 28-hpf *ubtor* mutants (Fig. 5D). An ISH experiment showed that rapamycin reduced the intensity of expression and number of *c-fos*-positive spinal interneurons in *ubtor* mutants (Fig. 5E–G). These results suggested that rapamycin inhibits the abnormally up-regulated activity of spinal interneurons in *ubtor*-mutated zebrafish, but has no detectable effect on the wild-type control group. They also suggest that the activation of mTORC1 pathway activity in *ubtor* mutants results in the up-regulated activity of spinal interneurons. Taken together, these studies indicate that the depletion of *ubtor* regulates the activity of the mTORC1 signaling pathway, thereby affecting the activity of neurons, and ultimately controlling the spontaneous movement of zebrafish embryos.

### The *Ubtor* Mutant Shows Increased Sensitivity to PTZ

**Fig. 6:**
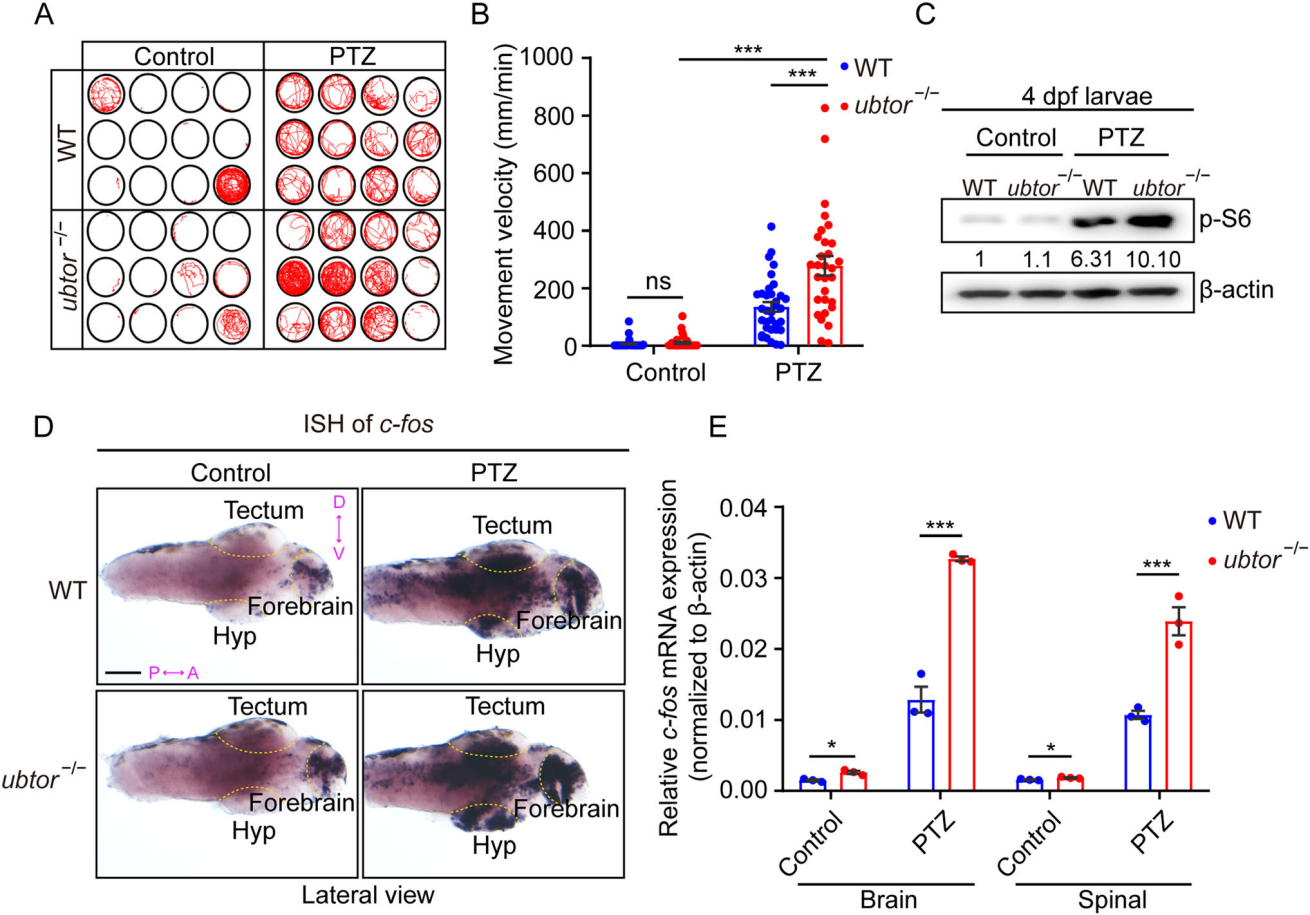
*ubtor* mutants exhibit hypersensitivity to PTZ. **A**, **B** Locomotion tracks **A** and movement velocity **B** of 4-dpf larvae treated with water (solvent) (three biological repeats, N_WT_ = N_*ubtor*_^−/−^ = 36, *t*_128_ = 0.737) or 3 mmol/L PTZ (three biological repeats, N_WT_ = N_*ubtor*_^−/−^ = 36, *t*_128_ = 4.753) in a 10-min period. **C** Immunoblotting for p-S6 protein in 4-dpf larvae treated with water or PTZ. Values of p-S6 protein levels are indicated. **D** Expression and distribution of *c-fos* mRNA in brain tissue of 4-dpf larvae treated with solvent (water) or PTZ (scale bar, 100 μm). **E** RT-qPCR analysis of *c-fos* mRNA levels in brain and spinal tissues of 4-dpf larvae treated with control or PTZ (three biological repeats, control: N_WT_ = N_*ubtor*_^−/−^ = 75; 3 mmol/L PTZ:N_WT_ = N_*ubtor*_^−/−^ = 75). β-actin served as the internal control. Values are represented as the mean ± SEM in **B** and **E**. *P <0.05, ***P <0.001.

The results above showed that *ubtor* depletion induces abnormal motor behaviors and neuronal activity, suggesting that *ubtor* mutations are involved in epilepsy-like behaviors in zebrafish. PTZ is a GABA receptor inhibitor that increases neuronal activity through the inhibition of GABA signaling networks. PTZ is also a common inducer in kindling models in mice, juvenile models, and adult zebrafish models of epilepsy [25, 31]. We therefore used PTZ to induce epilepsy-like behavior in *ubtor* mutant zebrafish and wild-type controls at 4 dpf. Prior to the PTZ treatment, both control and *ubtor* mutant 4-dpf larvae had low activity levels (*ubtor* mutant: 42.77 ± 17.68 mm/min *vs* control: 21.28 ± 13.21 mm/min, *P* = 0.3336, Fig. 6A, B). After PTZ treatment, both the wild-type and *ubtor* mutant had significantly increased activity levels. Strikingly, the motor activity of the *ubtor* mutant was significantly higher than that of the wild-type control (*ubtor* mutant: 277.6 ± 33.65 mm/min *vs* control: 134.5 ± 16.88 mm/min, *P* <0.001, Fig. 6A, B). To assess mTORC1 activation after PTZ induction, we used a p-S6 Western blot. Before PTZ treatment, both the wild-type and *ubtor* mutant at 4 dpf had low levels of p-S6 protein. After PTZ induction, the p-S6 protein levels were up-regulated both in the wild-type and *ubtor* mutant, with a stronger up-regulation in the mutant (Fig. 6C). A similar phenomenon was found with regard to *c-fos* expression in the brain and spinal tissues (the latter included muscle tissue surrounding the spinal cord) in response to PTZ treatment in the wild-type and *ubtor* mutant at 4 and 14 dpf (Fig. 6D, E and S5). These results indicated that the *ubtor* mutant zebrafish are more prone to motor hyperactivity after treatment with PTZ, along with activation of the mTOR signaling pathway and higher neuronal activity in the brain and spinal cord.

### Rapamycin Rescues Seizure Susceptibility Through PTZ-Induction

**Fig. 7:**
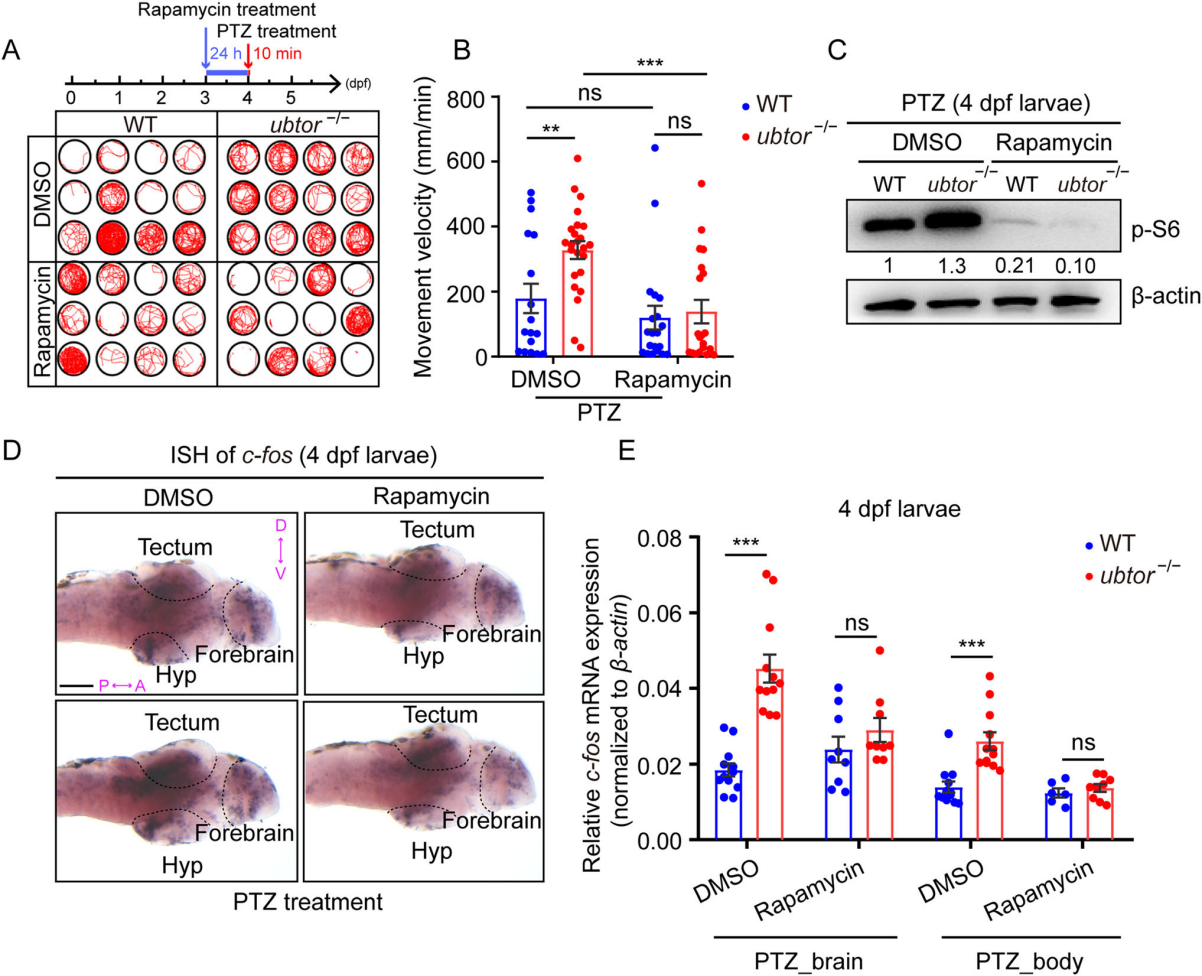
Rapamycin rescues seizure susceptibility in PTZ-induced *ubtor* mutant larvae. **A**, **B** Locomotion tracks **A** and movement velocity **B** of 4-dpf larvae pre-treated with DMSO (N_WT_ = N_*ubtor*_^−/−^ = 24, *t*_39_ = 2.987) or 2 µmol/L rapamycin (N_WT_ = N_*ubtor*_^−/−^ = 24, *t*_38_ = 0.365), followed by 3 mmol/L PTZ (two biological repeats).**C** Immunoblotting for p-S6 protein in 4-dpf larvae treated as in A. Values of p-S6 protein levels are indicated. **D** Expression and distribution of *c-fos* mRNA in brain tissue of 4-dpf larvae treated with rapamycin and PTZ (scale bar, 100 μm). **E** RT-qPCR analysis of *c-fos* mRNA levels in the brain and spinal tissues of 4-dpf larvae treated with control or rapamycin, followed by 3 mmol/L PTZ (three biological repeats, control: N_WT_ = N_*ubtor*_^−/−^ = 75; rapamycin: N_WT_ = N_*ubtor*_^−/−^ = 75). β-actin served as the internal control. Values are represented as the mean ± SEM in **B** and **E**. **P <0.01, ***P <0.001.

Studies have shown that rapamycin inhibits the occurrence and development of seizures and reduces their frequency in several models of epilepsy [32]. Therefore, we next examined whether rapamycin could rescue the seizure susceptibility induced by PTZ exposure in *ubtor* mutants. At 4 dpf, the subject group (treated with rapamycin at 3 dpf) and the control group were exposed to PTZ and their motor behaviors were recorded (Fig. 7A, B). The results showed that rapamycin treatment significantly rescued the amount of movement by *ubtor* mutants after PTZ exposure, reaching levels similar to those of the wild-type control (*ubtor* mutant: 135.4 ± 36.11 mm/min *vs* control: 116.6 ± 36.95 mm/min, *P* = 0.717, Fig. 7A, B). After the behavioral experiments, we extracted the total protein of 4-dpf larvae and analyzed the p-S6 protein levels to determine the activity of the mTORC1 signaling pathway. Compared with the DMSO control group, rapamycin treatment significantly decreased the p-S6 protein levels both in wild-type and *ubtor*-mutated zebrafish (Fig. 7C). These results suggested that rapamycin inhibits the PTZ-induced mTORC1 pathway activity in *ubtor* mutants. ISH and RT-qPCR experiments also showed that rapamycin pretreatment rescued the up-regulated expression of *c-fos* in *ubtor* mutants after PTZ exposure (Fig. 7D, E).

## Discussion

In this study, we showed that the spontaneous movements of *ubtor* mutant zebrafish were increased compared with the wild-type control group, and neuronal activity in the *ubtor* mutant was elevated. Mechanistically, we demonstrated that mTOR signaling was increased in the *ubtor* mutant, and the mTORC1 inhibitor rapamycin decreased the movements and neuronal activity in the mutant. These results suggested that *ubtor* regulates motor hyperactivity and epilepsy-like behaviors through mTOR signaling.

*UBTOR/KIAA1024/MINAR1* is a poorly-characterized gene. Our recent study showed that *Ubtor* participates in a novel negative feedback mechanism to control mTOR signaling [10]. It has also been suggested that *UBTOR/MINAR1* is implicated in Notch signaling and angiogenesis [13]. The *in vivo* function of *UBTOR* is largely unknown, except as a candidate gene in various cancers, and it has also been reported in genetic studies of neurodevelopmental disorders and intellectual disability [33]. Our results in this study showed that disruption of *ubtor* in zebrafish resulted in motor hyperactivity and elevated neuronal activity. These results established a role of *ubtor* in the nervous system *in vivo*. This role is also consistent with the strong *ubtor* expression in the central nervous system in vertebrates including fish, mice, and humans.

Loss of mTOR-negative regulators and over-activation of the mTOR signaling pathway are major causes underlying the occurrence of cortical malformations and epileptic encephalopathy. For instance, *DEPDC5*, a component of the GATOR1 complex and a negative regulator of mTOR, has been identified as a gene associated with focal epilepsy in humans [34]. In zebrafish, *depdc5* mutant larvae show mTOR hyperactivation, epileptic discharges in the brain, and increased motor hyperactivity in response to PTZ treatment [9, 35]. Thus, the phenotypic abnormalities found in the *depdc5* mutant are similar our findings in the *ubtor* mutant larvae. Both the *depec5* and *ubtor* mutation studies in zebrafish suggest that hyperactivation of mTOR signaling disrupts the balance between excitatory and inhibitory activity, which leads to abnormalities in neural functions. Our results showed that treatment with the mTORC1 inhibitor rapamycin rescued the motor hyperactivity and the elevated neuronal activity in the *ubtor* mutant, indicating that mTOR signaling is the major cause underlying the phenotypic abnormalities in the *ubtor* mutant. Treatment with rapamycin was also effective in reversing the phenotypic abnormalities found in the *depdc5* mutant [9, 35], and in another larval mutant gene, *tsc2*, an mTOR-negative regulator [36]**.**

Zebrafish embryos in early development have relatively simple motor behavior and neural circuits, which can exhibit abnormal neural circuit development or activity as a result of genetic mutations. We found that the spinal interneurons of 28-hpf *ubtor* mutants developed normally, while *c-fos* gene expression was significantly up-regulated in spinal neurons. This result suggests that the activity of *ubtor* mutant spinal neurons is up-regulated. However, the specific types of spinal interneurons involved in *ubtor* mutants need to be identified in further studies. We further used Ca^2+^ imaging to examine the activity changes of spinal neurons at higher spatial and temporal resolution. The results showed that the neuronal activity of CiD spinal interneuron was significantly increased in *ubtor* mutants. Previous studies have shown that CiD interneurons express the excitatory neurotransmitter glutamate and function as premotor interneurons to drive the generation of motor rhythms in larvae at 52 hpf or older [37–39]. In contrast, *in vivo* patch clamp electrophysiological recordings in zebrafish embryonic spinal cord neurons has shown that, at 20–24 hpf, the three primary interneurons IC (ipsilateral caudal), VeLD (ventral longitudinal descending), and CoPA are active when the embryonic trunk contracts spontaneously, while the four types of secondary interneurons, CiA (circumferential ascending), CiD (circumferential descending), CoBL (commissural bifurcating longitudinal), and CoSA, show no evidence of significant activity in the context of spontaneous behaviors [22]. This difference may be due to the fact that the axons of the secondary interneurons extend later than those of the primary interneurons and may have not yet projected to the motor neurons that drive motor behaviors [40]. The time point of our examination of spinal interneuron activity was at 28 hpf, and we saw strong and robust neuronal activity only in CiD neurons. Future experiments applying subtype-specific transgenic lines, voltage-sensitive reporters [41], and electrophysiological examination of these spinal interneurons are required to elucidate the exact roles of *Ubtor* in the regulation of spinal circuit activity.

Our results here showed that *ubtor* mutant larvae were hypersensitive to PTZ. PTZ is widely used to chemically induce seizures in mouse and zebrafish models [25, 42]. The neuropathological effects of PTZ treatment are complex; it alters both GABA-mediated inhibition and glutamate-mediated excitation [43]. Because the consequences of PTZ treatment for the *ubtor* mutant were highly significant, it is likely their effects were synergistic. Further examination of mTOR signaling activity and *c-fos* expression suggested that disruption of the *ubtor* gene enhances the sensitivity of PTZ-induced epilepsy-like behaviors by up-regulating mTORC1 pathway activity. Strikingly, rapamycin treatment restored the increased *c-fos* expression and swimming activity of *ubtor* mutants exposed to PTZ. Recent research has indicated that treatment with rapamycin for a period of time prior to spontaneous epilepsy or drug-induced seizures inhibits seizures or reduces their severity, which may be related to inhibition of neuronal excitability and neurotransmitter release [30, 35]. These results further support the hypothesis that the *ubtor* mutation participates in the regulation of epilepsy-like behaviors through activation of the zebrafish mTORC1 pathway, thereby up-regulating neuronal activity. In future, it will be necessary to investigate how the enhancement of mTORC1 signaling caused by *ubtor* mutations modulated the up-regulation of neuronal activity, and to elucidate the mechanisms linked to the *ubtor*–mTOR–neuronal activity axis in the zebrafish model.

Taken together, our studies found that the *ubtor* gene plays a role in early spontaneous movement and epilepsy-like behaviors in zebrafish by regulating the mTOR signaling pathway and spinal interneuron activity. These salient features in the *ubtor* mutant zebrafish may also be used as a preclinical model for studies and screening for compounds regulating mTOR signaling and treating epilepsy.

## Supplementary Information

Below is the link to the electronic supplementary material.

Supplementary file1 (PDF 555 KB)
