# Supplementary material for: ubtor Mutation Causes Motor Hyperactivity by Activating mTOR Signaling in Zebrafish
Source: Neurosci Bull. 2021 Jul 26;37(12):1658–70. doi: 10.1007/s12264-021-00755-z (PMC8643380; doi:10.1007/s12264-021-00755-z)
Supplement: Supplementary file 1 — Supplementary file1 (PDF 555 KB) [file 12264_2021_755_MOESM1_ESM.pdf]

Supplementary Materials

Supplementary Figures

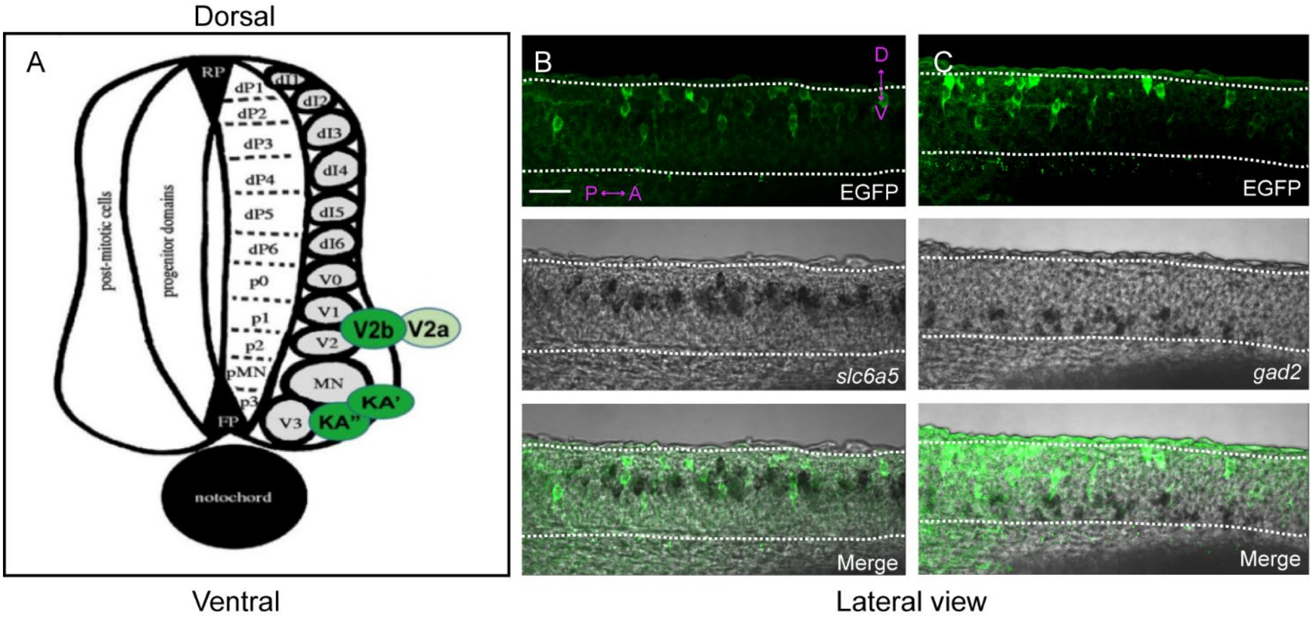

Fig. S1 Distribution of *ubtor*:EGFP and spinal neuron markers. **A** Model of stereotypically-arranged spinal neurons along the dorsal-ventral axis of the spinal cord. Modified from [1]. MN, motor neurons; RP, roof plate; FP, floor plate; KA, Kolmer-Agduhr neurons. **B** Immunofluorescence of EGFP and *in situ* hybridization of *slc6a5*/glyT2. **C** Immunofluorescence of EGFP and *in situ* hybridization of *gad2*. D, dorsal; V, ventral; P, posterior; A, anterior. Scale bar, 60 μm.

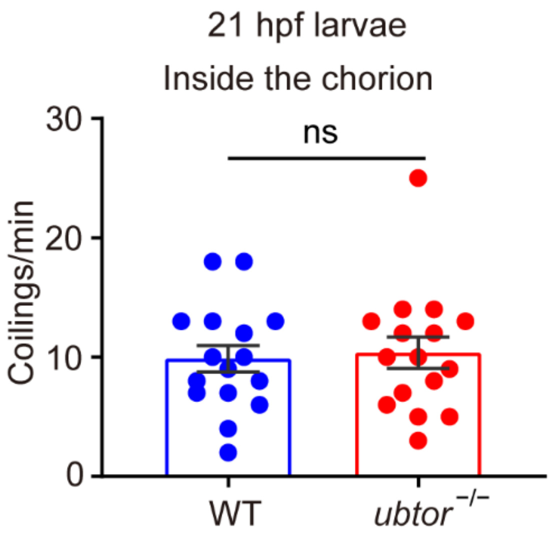

Fig. S2 Frequency of coiling movements of embryos inside the chorion of 21-hpf *ubtor* mutants or wild-type (WT) zebrafish ($N_{WT} = N_{ubtor^{-/-}} = 16$, $t_{30} = 0.2904$). Values are represented as the mean ± SEM. ns, not significant.

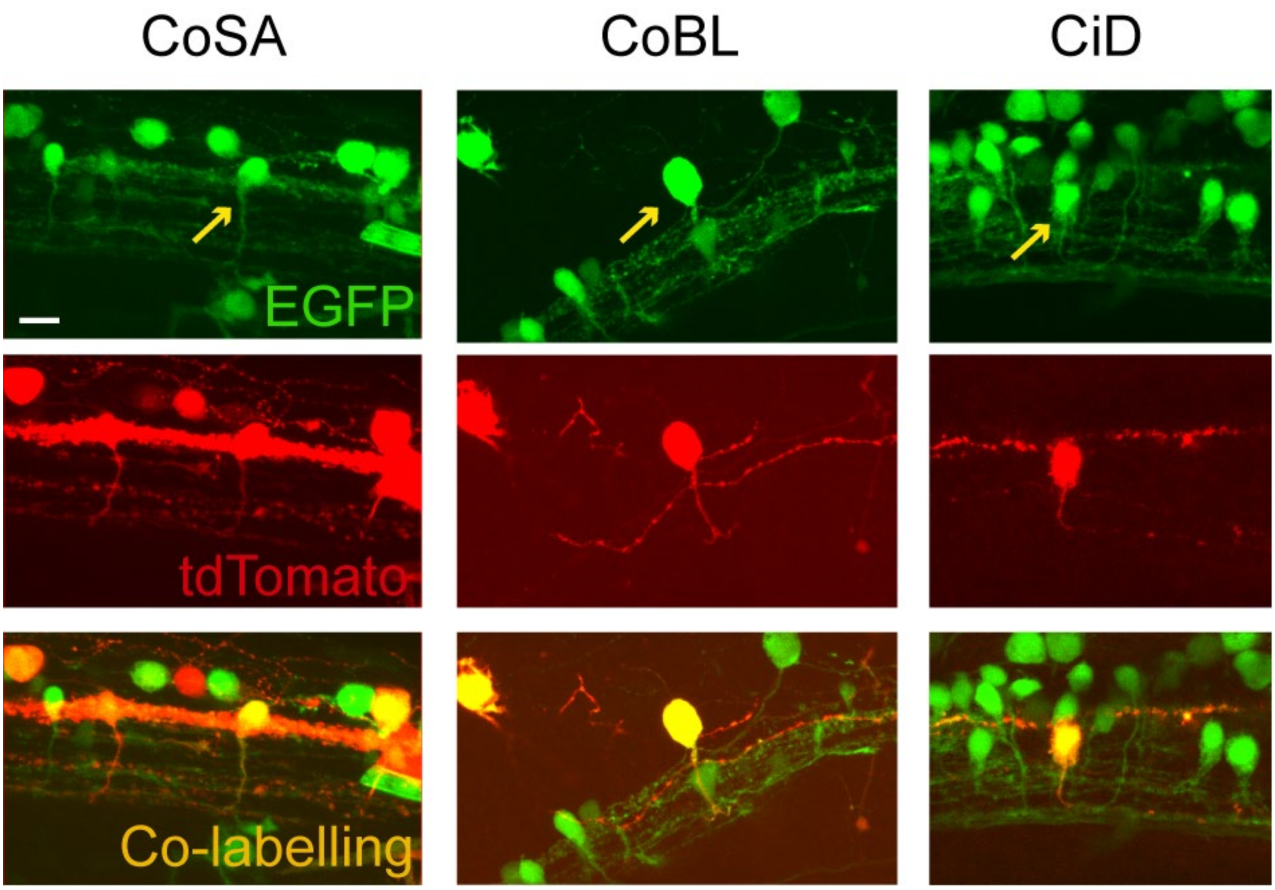

Fig. S3 Mosaic labelling of spinal interneurons. The mosaic labelling of CoSA, CoBL, and CiD interneurons by tdTomato in 28-hpf Tg(*ubtor*:GAL4;UAS:EGFP) transgenic embryos (scale bar, 20 μm; arrows indicate mosaic labelling of interneurons; CoSA, commissural secondary; CoBL, commissural bifurcating longitudinal; CiD, circumferential descending).

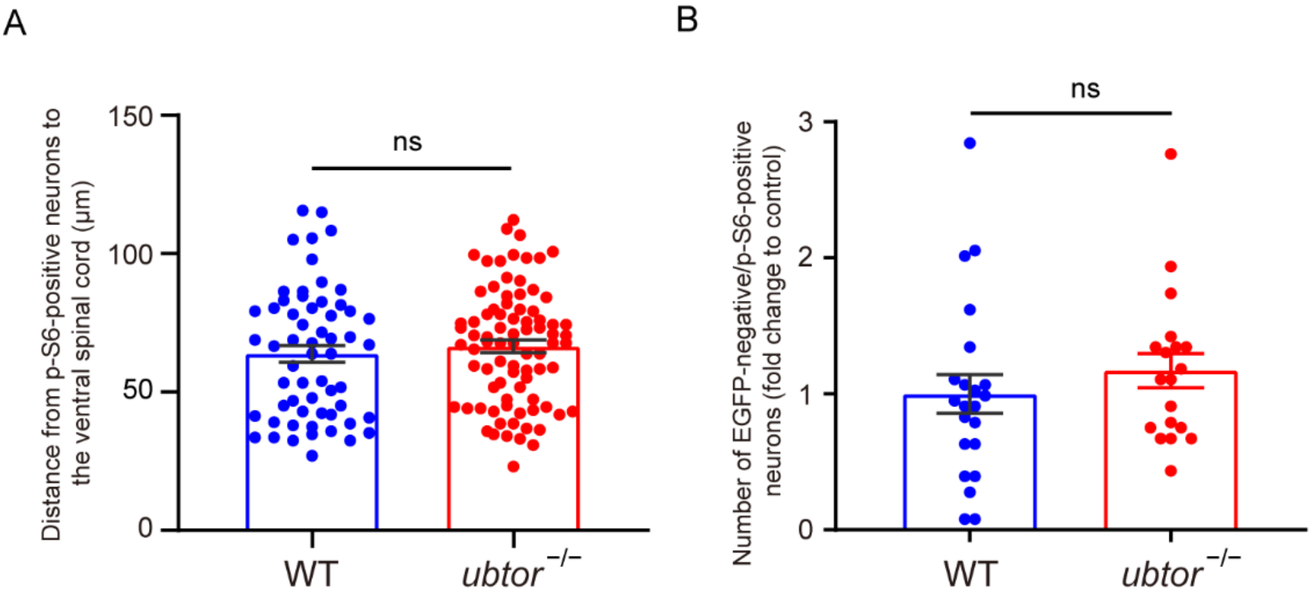

Fig. S4 Distribution of p-S6-positive cells along the dorsal-ventral axis of the spinal cord. **A** Distances from p-S6-positive neurons to the floor plate in the ventral spinal cord of 28-hpf WT and *ubtor* mutant zebrafish (p-S6-positive neurons: $N_{WT} = 59$, $N_{ubtor^{-/-}} = 83$, $t_{140} = 0.71$, $P = 0.479$). **B** Numbers of EGFP-negative/p-S6-positive neurons in the spinal cord of 28-hpf WT and *ubtor* mutant zebrafish (results from three biological repeats, $N_{WT} = 22$, $N_{ubtor^{-/-}} = 19$, $t_{39} = 0.8813$, $P = 0.384$). Values are represented as the mean ± SEM in **A** and **B**. ns, not significant.

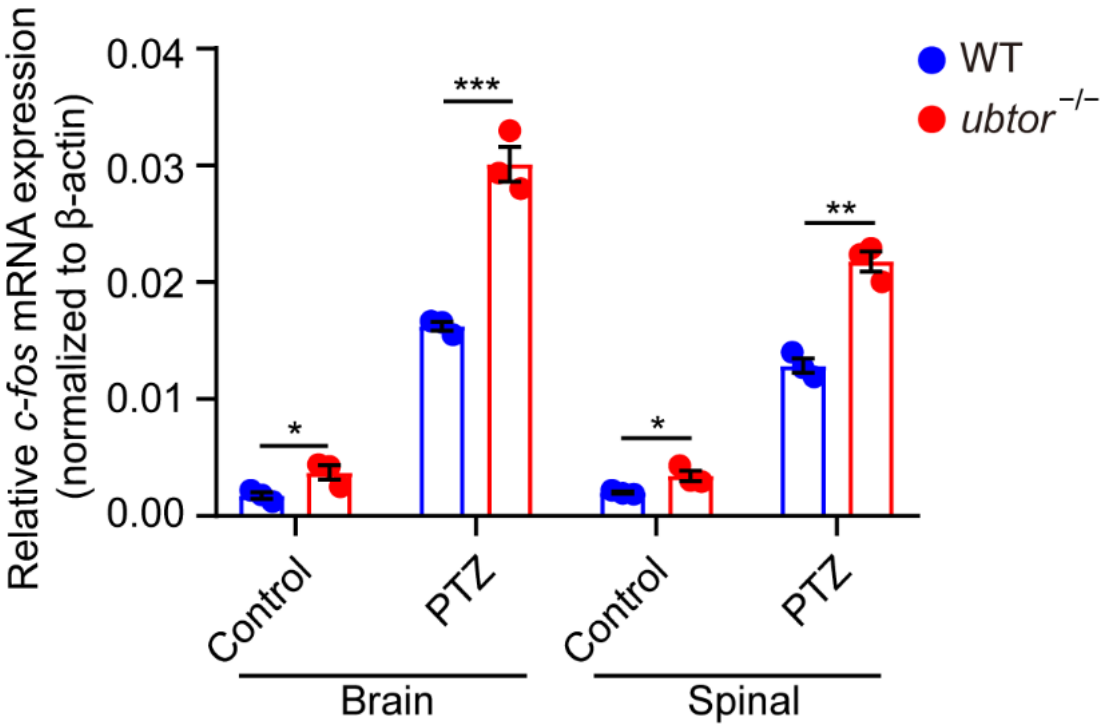

Fig. S5 The *c-fos* expression levels in the brain and spinal tissues of 14-dpf *ubtor* mutant and WT zebrafish before and after PTZ exposure (two biological repeats, control: $N_{WT} = N_{ubtor^{-/-}} = 48$; PTZ: $N_{WT} = N_{ubtor^{-/-}} = 48$). β-actin served as internal control. Values are represented as the mean ± SEM. *P <0.05, **P <0.01, ***P <0.001.

References

[1] Lewis KE. How do genes regulate simple behaviours? Understanding how different neurons in the vertebrate spinal cord are genetically specified. Philos Trans R Soc Lond B Biol Sci 2006, 361: 45–66.
